# Supplementary material for: The causal relationship between the human gut microbiota and pyogenic arthritis: a Mendelian randomization study
Source: Front Cell Infect Microbiol. 2024 Nov 26;14:1452480. doi: 10.3389/fcimb.2024.1452480 (PMC11629706; doi:10.3389/fcimb.2024.1452480)
Supplement: Supplementary file 1 [file DataSheet1.zip › mendelian test/ebi-a-GCST90027839.csv_forest.pdf]

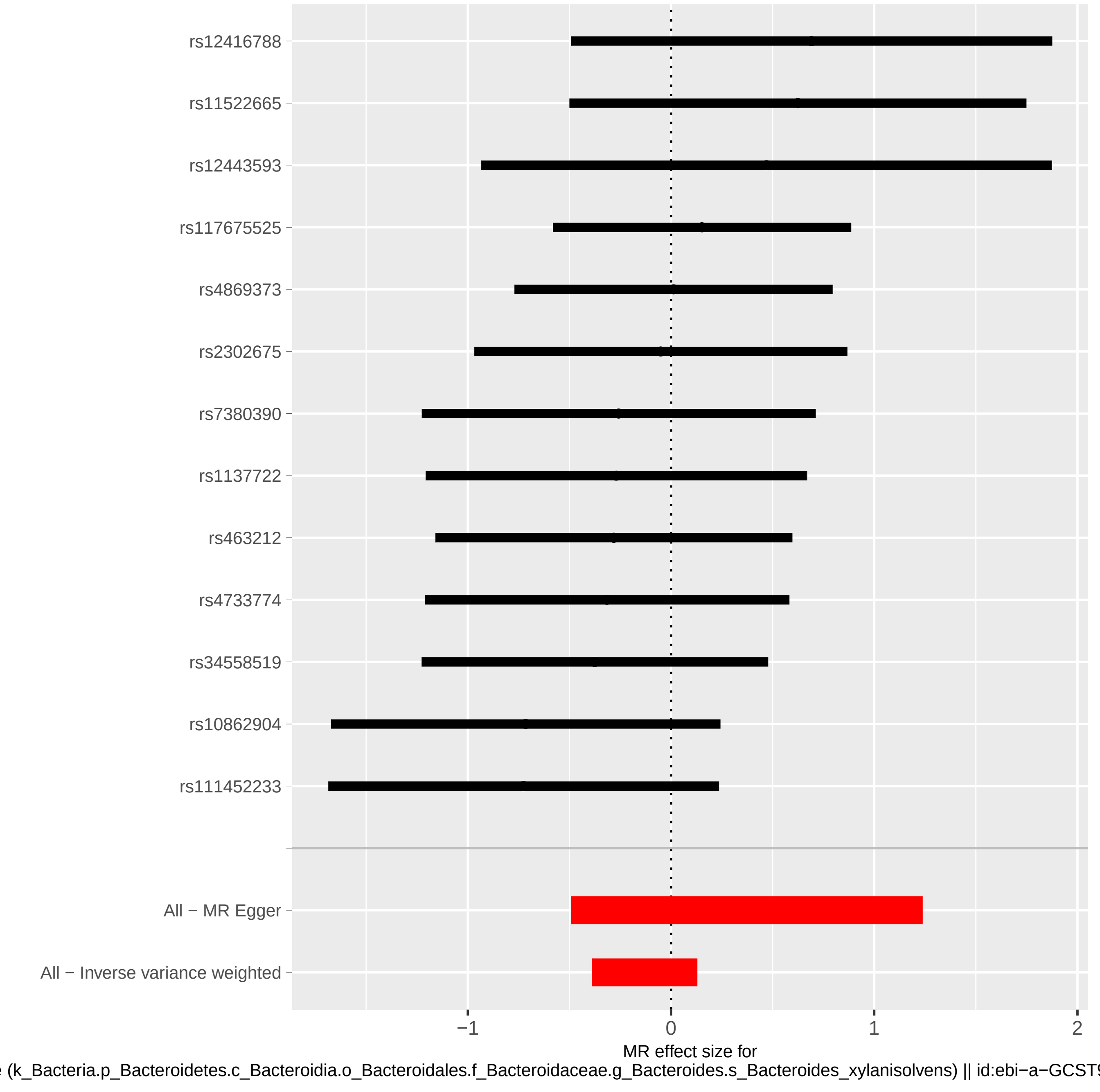

rs12416788
rs11522665
rs12443593
rs117675525
rs4869373
rs2302675
rs7380390
rs1137722
rs463212
rs4733774
rs34558519
rs10862904
rs111452233
All − MR Egger
All − Inverse variance weighted
−1
0
1
2
MR effect size for
e (k_Bacteria.p_Bacteroidetes.c_Bacteroidia.o_Bacteroidales.f_Bacteroidaceae.g_Bacteroides.s_Bacteroides_xylanisolvens) || id:ebi−a−GCST9
